# A Novel Voice Sensor for the Detection of Speech Signals

**DOI:** 10.3390/s131216533

**Published:** 2013-12-02

**Authors:** Kun-Ching Wang

**Affiliations:** Department of Information Technology & Communication, Shih Chien University, 200 University Road, Neimen, Kaohsiung 84550, Taiwan; E-Mail: kunching@mail.kh.usc.edu.tw; Tel.: +886-07-667-8888-5723; Fax: +886-07-667-8888-4332

**Keywords:** voice sensor, two-dimensional, part-band energy entropy, long-term information analysis, Mel-scaled filter bank

## Abstract

In order to develop a novel voice sensor to detect human voices, the use of features which are more robust to noise is an important issue. Voice sensor is also called voice activity detection (VAD). Due to that the inherent nature of the formant structure only occurred on the speech spectrogram (well-known as voiceprint), Wu *et al.* were the first to use band-spectral entropy (BSE) to describe the characteristics of voiceprints. However, the performance of VAD based on BSE feature was degraded in colored noise (or voiceprint-like noise) environments. In order to solve this problem, we propose the two-dimensional part-band energy entropy (TD-PBEE) parameter based on two variables: part-band partition number upon frequency index and long-term window size upon time index to further improve the BSE-based VAD algorithm. The two variables can efficiently represent the characteristics of voiceprints on each critical frequency band and use long-term information for noisy speech spectrograms, respectively. The TD-PBEE parameter can be regarded as a PBEE parameter over time. First, the strength of voiceprints can be partly enhanced by using four entropies applied to four part-bands. We can use the four part-band energy entropies for describing the voiceprints in detail. Due to the characteristics of non-stationary for speech and various noises, we will then use long-term information processing to refine the PBEE, so the voice-like noise can be distinguished from noisy speech through the concept of PBEE with long-term information. Our experiments show that the proposed feature extraction with the TD-PBEE parameter is quite insensitive to background noise. The proposed TD-PBEE-based VAD algorithm is evaluated for four types of noises and five signal-to-noise ratio (SNR) levels. We find that the accuracy of the proposed TD-PBEE-based VAD algorithm averaged over all noises and all SNR levels is better than that of other considered VAD algorithms.

## 1. Introduction

So far, user-friendly voice interfaces have been widely used in consumer devices, such as interactive digital TV, personal digital assistants and cellular phones [1–3]. Voice sensor (also called voice activity detection, VAD) refers to the problem of distinguishing speech from non-speech regions. It is found that VAD is a critical component in voice-command application. However, the use of features which are more robust to noise is an important issue. Various types of different approaches to VAD have been proposed recently. In early VAD algorithm designs, short-term energy, zero-crossing rate and LPC coefficients [4] were used as feature parameters for detecting voices. In addition, some algorithms further used cepstral features [5], formant shape [6], and least-square periodicity measures [7]. Others have used correlation coefficients [8], wavelet coefficients [9], entropy measures [10], and a set of metrics [11]. Remirez *et al.* recently formulated long-term spectral divergence (LTSD) between speech and non-speech segments as a discriminative speech feature [12]. Ma *et al.* further proposed a long-term spectral flatness measure (LSFM) to improve speech detection robustness for lower SNR [13]. More complex algorithms use statistical model-based features [14,15], which have decision rules derived from the likelihood ratio test.

In fact, a robust VAD algorithm in the presence of different types of noises is necessary and critical. Depending on the characteristics of the human voice, a variety of parameters has been proposed for VAD. In general, no particular feature or specific set of features has been shown to perform uniformly well under different noise conditions. For example, energy-based features do not work well at low SNR [16]. Similarly, entropy measures fail to distinguish speech from noise with good accuracy due to the colored spectrum of speech [17]. SNR estimation is also a critical component in many of the existing VAD schemes, which is particularly difficult for non-stationary noise [18]. The use of features which are more robust to noise is an important issue for develop a robust VAD algorithm. Due to the fact that the inherent nature of the formant structure only occurred on speech spectrograms and is the well-known as the “voiceprint”, Wu *et al.* were the first to use band-spectral entropy (BSE) to describe the characteristics of voiceprints [19]. However, the performance of BSE-based features for VAD was degraded under colored noise environment conditions.

In order to solve this problem, we propose a two-dimensional part-band energy entropy (TD-PBEE) method in this paper to improve the robustness of the proposed VAD method in colored noisy environments. The TD-PBEE parameter can be regarded as the relation of spectral entropy *versus* time index. In summary, the TD-PBEE is based on two variables: part-band number (*N*) upon frequency index and long-term size (*R*) upon time index. First, the four part-bands (the optimal is *N* = 4) derived from 17 log-energies by a Mel-scaled filter bank are partitioned as a lowest frequency (1–8 Mel) part, a low frequency (9–12 Mel) part, a high frequency (13–15 Mel) part and a highest frequency (16–17 Mel) part. Consequently, the strength of voiceprints can be more enhanced by four PBEEs than that by BSE. Secondly, we will use long-term information processing to refine the PBEE due to the non-stationary characteristics of speech and various noises. Each part-band has different long-term window *R* sizes. Through different *R* values, the TD-PBEE dependent on each part-band will be determined to efficiently represent the voiceprint characteristics in each critical frequency band. Consequently, the voice-like noise can be distinguished from noisy speech through the concept of PBEE with long-term information. Our experiments show that the proposed feature extraction of TD-PBEE is quite insensitive to background noise. The proposed TD-PBEE-based VAD scheme is evaluated for four types of noises and five signal-to-noise ratio (SNR) levels. We find that the accuracy of the proposed TD-PBEE-based VAD method averaged over all noise and all SNR levels is better than that of other considered VAD algorithms.

The remainder of this paper is organized as follows: in Section 2, the procedure of determining the TD-PBEE parameter is described. In Section 3, the proposed VAD based on TD-PBEE is schematically introduced. In Section 4, experimental results demonstrate the effectiveness of the proposed TD-PBEE VAD method. Finally, Section 5 concludes the paper.

## 2. The Proposed Two-Dimensional Part-Band Energy Entropy (TD-PBEE) Measure

**Figure 1.**
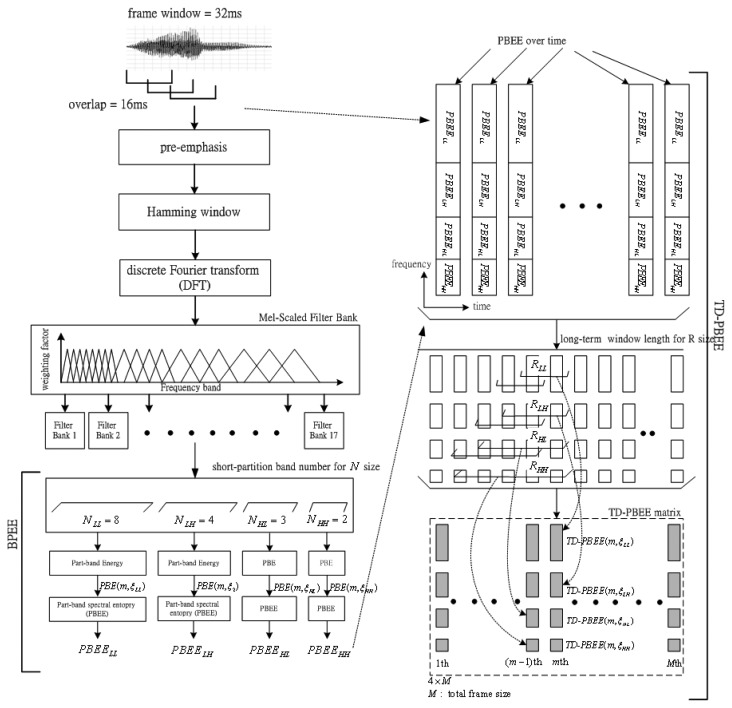
The block diagram of the feature extraction for TD-PBEE measurements.

According to the findings from [18], Wu *et al.* were the first to use BSE to describe the voiceprint characteristics of speech-only spectrograms. It is found that the BSE can detect the human-voice signals. In this subsection, we further improve the BSE and propose a novel feature extraction of the TD-PBEE parameter. The definition of the TD-PBEE will be shown in detail. Figure 1 shows the procedure of feature extraction of TD-PBEE. Observing Figure 1, we can find the procedure of the TD-PBEE is based on (*R*, *N*). The input speech signal is frame windowed (32-ms length and 16-ms shift) using the Hamming window. In order to spectrally flatten the signal and to make it less susceptible to finite precision effects later in the signal processing, the digitized speech signal is first put through a first-order pre-emphasis filter with pre-emphasis coefficient 0.97:

(1) $$H(z)=1-0.97z^{-1}$$

After the pre-emphasis process, a speech signal is divided into frames by multiplying a Hamming window. In order to avoid sharp changes, we make the windows overlap with each other. Hence, the utterance is segmented into a sequence of overlapped frames. Secondly, a Discrete Fourier Transform (DFT) is applied to obtain the short time spectrum of each frame. We then multiply the spectrum by the common Mel-scale filter bank weighting factors and compute the energy of each frequency band. We generate the output energy of each filter of the 17-channel Mel-scale filter bank. Then, the short-partition band number, *N*, is used in the paper. The value of *N* is four and comprises a set of *N_LL_*, *N_LH_* and *N_HH_* (*N* = { *N_LL_*, *N_LH_*, *N_HL_*, *N_HH_* }), so the four part-band energy (PBE) is denoted as *PBE*(*m*, *η_LL_*), *PBE*(*m*, *η_LH_*), *PBE*(*m*, *η_HL_*) and *PBE*(*m*, *η_HH_*). Each of the short-partition bands shows a lowest frequency (1–8 Mel) part (*N_LL_*=8), a low frequency (9–12 Mel) part (*N_LH_*=4), a high frequency (13–15 Mel) part (*N_HL_*=3) and a highest frequency (16–17 Mel) part (*N_HH_*=2). The strength of voiceprints can be partly enhanced by using four part-band energy entropies (PBEE) applied to the four part-bands. Consequently, the voiceprint-like noise will not be detected in noisy speech. The inherent characteristic of voiceprints can be better characterized by PBEE than by the BSE parameter.

Finally, collecting a sequence of PBEE coefficients along the time axis, we can get a PBEE over time. Applying the long-term spectral information processing for R size, the value of each TD-PBEE is depended on different *R* : (*R_LL_*, *R_LH_*, *R_HL_* and *R_HH_*) The value of *PBEE_LL_* over *R_LL_* consecutive frames is determined at the specific *LL*th part-band. Similarly, the value of *PBEE_LH_* is determined over *R_LH_* consecutive frames at the specific *LH*th part-band. The value of *PBEE_HL_* is determined over *R_HL_* consecutive frames at the specific *HL*th part-band. The value of *PBEE_HH_* is determined over *R_HH_* consecutive frames at the specific *HH*th part-band. Consequently, the TD-PBEE parameters are chosen from the set of *TD-PBEE_LL_*, *TD-PBEE_LH_*, *TD-PBEE_HL_* and *TD-PBEE_HH_* coefficients over long-term average processing. The TD-PBEE parameter can be regarded as the relation of spectral entropy *versus* time index, so we also call it the TD-PBEE matrix. In this section, we will first introduce the definition of the PBEE based on *N*. Then, the TD-PBEE based on *R* will be presented later.

### 2.1. Definition of the PBEE Based on N

In order to further improve the advantage of characterizing voiceprints though band-spectral entropy (BSE), we adopt a novel concept of part-band spectral entropy (PBEE). This concept lets full-bands be partitioned into some little part-bands. Through spectral entropy determined from each part-band, the voiceprint can be more partially described.

**Figure 2.**
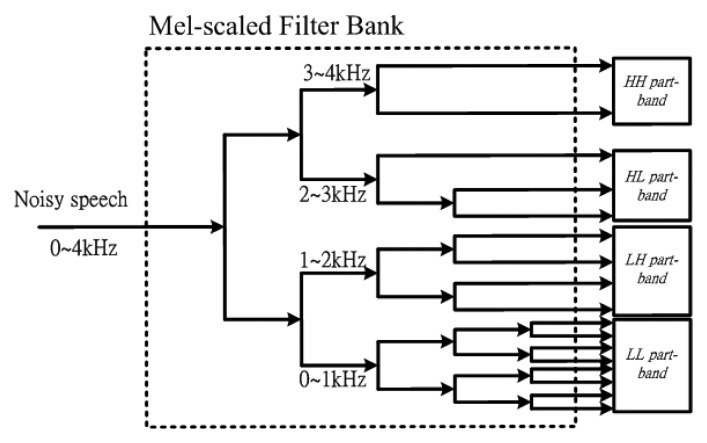
The structure of a four part-band partition.

Figure 2 shows the partition structure of the Mel-scaled filter bank. It is found that higher sub-band numbers are focused on the lower frequencies. Inversely, the lower sub-band numbers are focused on higher frequencies. Observing the Figure 2, each part-band has a different band number. Although many part-band numbers can clearly describe the voiceprint, this will need more computer power. In Table 1, we observe the fact that a higher number of part-band partitions can achieve higher VAD accuracy, but we need more computing time to run the VAD algorithm. Inversely, a lesser number of part-band partitions leads to lower VAD accuracy. Considering the trade-off between accuracy and real-time requirements, the number of part-band partitions, *N* equal four is best compromise. The numbers of each part-band are *N_LL_* = 8, *N_LH_* = 4, *N_HL_* = 3 and *N_HH_* = 2, respectively. The four part-bands comprise 0∼1 kHz (LL part-band *η_LL_* : 1–8 Mel), 1∼2 kHz (LH part- band *η_LH_* : 9–12 Mel), 2∼3 kHz (HL part-band *η_HL_* : 13–15 Mel) and 3∼4 kHz (HH part-band *η_HH_* : 16–17 Mel). Consequently, the PBEE parameter at each part-band is computed as below:

(2) $$\text{PBEE}(m,\xi_{LL})=-\sum_{\xi =1}^{N_{LL}}P_{LL}(m,\xi )\log P_{LL}(m,\xi )$$

(3) $$\text{PBEE}(m,\xi_{LH})=-\sum_{\xi =N_{LL}+1}^{N_{LL}+N_{LH}}P_{LH}(m,\xi )\log P_{LH}(m,\xi )$$

(4) $$\text{PBEE}(m,\xi_{HL})=-\sum_{\xi =N_{LH}+1}^{N_{LH}+N_{HL}}P_{HL}(m,\xi )\log P_{HL}(m,\xi )$$

(5) $$\text{PBEE}(m,\xi_{HH})=-\sum_{\xi =N_{HL}+1}^{N_{HL}+N_{HH}}P_{HH}(m,\xi )\log P_{HH}(m,\xi )$$

where $P_{LL}(m,\xi )=\overset{E_{b}(m,\xi )}{\;}/\underset{\sum_{k=1}^{N_{LL}}E_{b}(m,k)}{\;}$ is the probability corresponding to 17 band-energies. Band-energy for each frame is denoted as *E_b_*(*m*, *ξ*) = |*X*(*m*, *ξ*)|^2^.

Similar, the other probabilities are defined as below:

(6) $$P_{LH}(m,\xi )=\overset{E_{b}(m,\xi )}{\;}/\underset{\sum_{k=N_{LL}+1}^{N_{LH}}E_{b}(m,k)}{\;}$$

(7) $$P_{HL}(m,\xi )=\overset{E_{b}(m,\xi )}{\;}/\underset{\sum_{k=N_{LH}+1}^{N_{HL}}E_{b}(m,k)}{\;}$$

(8) $$P_{HH}(m,\xi )=\overset{E_{b}(m,\xi )}{\;}/\underset{\sum_{k=N_{HL}+1}^{N_{HH}}E_{b}(m,k)}{\;}$$

**Figure 3.**
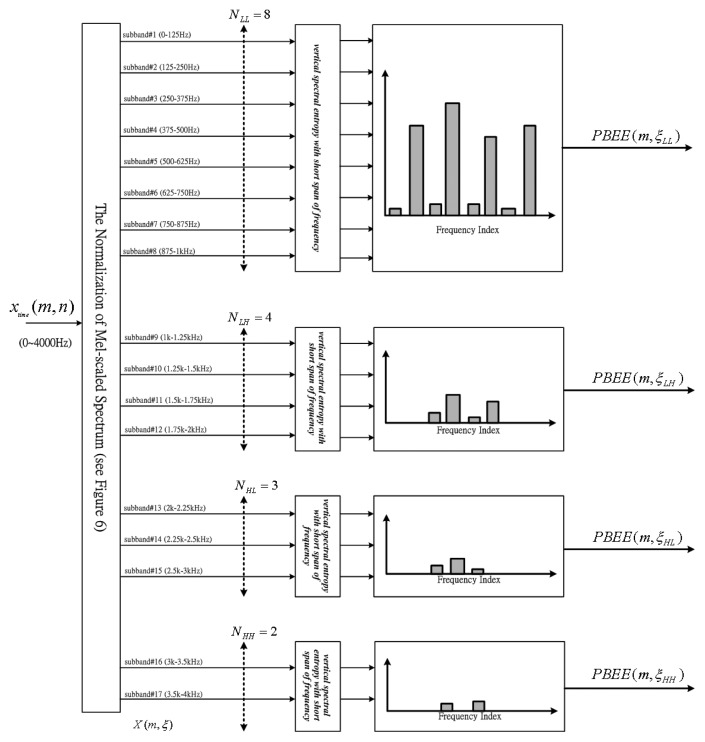
The block diagram of four PBEE values determined from four part-bands.

Figure 3 shows the four PBEE values determined from four part-bands. We can find that the PBEE value is dependent on the different frequency band numbers *N*. Due to the fact that the voiceprints mostly focus on middle or low frequency band, more band numbers are required. Inversely, less band numbers are assigned to the higher frequency band due to the fact that the higher band is almost always dominated by noise components.

### 2.2. The TD-PBEE Based on R

In order to further refine the PBEE parameter, long-term information processing is used to determine a reliable evaluation for the strength of voiceprint on part-band. In this subsection, each part-band has different long-term windows size corresponding to *R_LL_*, *R_LH_*, *R_HL_* and *R_HH_*. Due to the fact that voiceprint-like noise can often focus on high frequency bands, a concept of long-term information is required, so the assumption is expressed as *R_LL_* < *R_LH_* < *R_HL_* < *R_HH_* for four PBEE parameters of each part-band. In addition, this assumption also reduces the search time decreasing computing power for the low frequency band and increasing the accuracy of voiceprint evaluation for the entire speech signal.

Consequently, the definition of two dimensions for PEBB parameter means that the one dimension is the time index and the other dimension is the frequency index. The computation of the TD-PBEE is shown as below:

(9) $$TD-\text{PBEE}(m,\xi_{LL})=\sum_{n=m-R_{LL}}^{m}\text{PBEE}(n,\xi_{LL})/R_{LL}$$

(10) $$TD-\text{PBEE}(m,\xi_{LH})=\sum_{n=m-R_{LH}}^{m}\text{PBEE}(n,\xi_{LH})/R_{LH}$$

(11) $$TD-\text{PBEE}(m,\xi_{HL})=\sum_{n=m-R_{HL}}^{m}\text{PBEE}(n,\xi_{HL})/R_{HL}$$

(12) $$TD-\text{PBEE}(m,\xi_{HH})=\sum_{n=m-R_{HH}}^{m}\text{PBEE}(n,\xi_{HH})/R_{HH}$$

**Figure 4.**
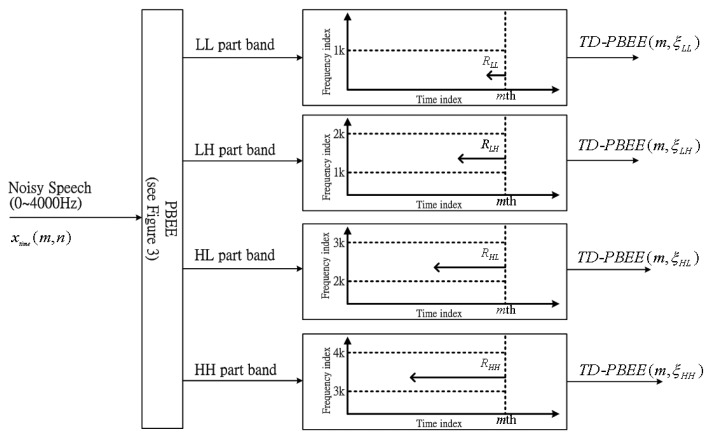
The block diagram of four TD-PBEE values determined from four PBEEs over time.

From the above equation can be found that each TD-PBEE is averaged over the long-term window size. Figure 4 clearly shows the block diagram of four TD-PBEE values determined from four PBEEs over time derived from different long-term window sizes: *R_LL_*, *R_LH_*, *R_HL_* and *R_HH_*.

## 3. The Proposed VAD Based on TD-PBEE Measure

**Figure 5.**
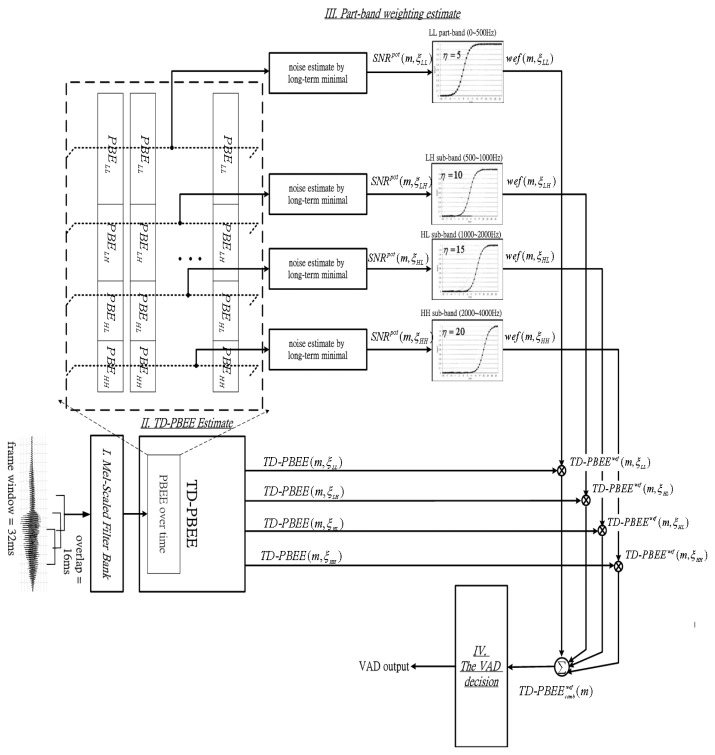
The block diagram of TD-PBEE based VAD algorithm.

In this section we propose the TD-PBEE based VAD algorithm as shown in Figure 5. The proposed TD-PBEE VAD method consists of four components: (1) Mel-scaled filter bank; (2) TD-PBEE estimate; (3) part-band weighting estimation; and (4) the VAD decision. TD-PBEE estimate has been introduced in Section 2. The remainder will be introduced in details as follows: first, the PBEE vector is applied to determine the part-band weighting estimate for suppressing voiceprints corrupted by noise. Secondly, we can use a part-band weighting estimate to adjust a robust TD-PBEE parameter. Finally, the VAD decision can adaptively judge whether the current frame is a noise-dominated frame or speech-dominated frame through a decision rules.

### 3.1. The Normalization of Mel-Scale Filter Bank

**Figure 6.**
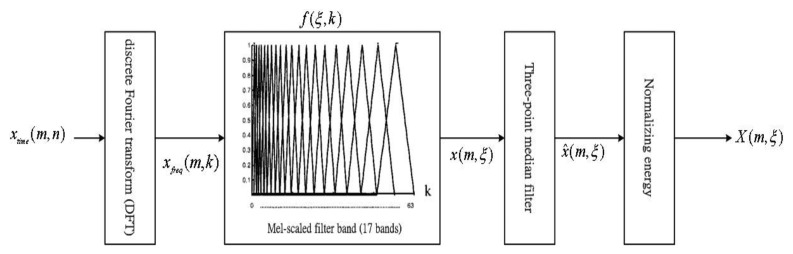
The processing of the Mel-scale filter bank.

Figure 6 shows in detail the process of including the Mel-scale bank for getting the normalized energy. The Mel-scale first suggested by Stevens and Volkman in 1937 [20] is a perceptually motivated scale. The scale was devised through human perception experiments where subjects were were asked to adjust a stimulus tone to perceptually half the pitch of a reference tone. Equation (1) is the Hz to Mel warping used in the experiments [21]:

(13) Mel=2595⋅log(1+f/700)

where *Mel* is the Mel-frequency scale and *f* is in Hertz. The filter banks of 17 bands are approximated by simulating 17 triangular bandpass filters, *f*(*η*,*k*) (1 ≤ *η* ≤ 17, 0 ≤ *k* ≤ 127), over a frequency range of 0–4 KHz. The energy of each frequency band for each time frame of a speech signal can be calculated through the Mel-scale frequency bank:

The spectrum, *x_freg_*(*m*,*k*), of this signal is first calculated by a Discrete Fourier Transform (256-point DFT), while considering a given time-domain noisy speech signal, *x_time_*(*m*,*n*), representing the magnitude of the *n*th point of the *m*th frame:

(14) $$\begin{array}{l} \begin{array}{c} x_{\text{freq}}(m,k)=\overset{N-1}{\underset{n=0}{\text{∑}}}x_{\text{time}}(m,n)\cdot \exp{\left(-j2\pi /N\right)}^{kn},0\leq k\leq N-1;\;0\leq m\leq M-1 \end{array} \end{array}$$

where *x_freg_*(*m*,*k*). means the magnitude of the *k*th point of the spectrum of the *m*th frame, and *M*. is the number of total frames of the speech signal for analysis.

First, the spectrum *x_freg_*(*m*,*k*) is then multiplied by the weighting factors *f*(*η*,*k*) on the Mel-scale frequency bank. Then, we can sum the products for all *_k_* to get the energy x(*m*,*η*) of each frequency band *η* of the *m*th frame:

(15) $$\begin{array}{l} \begin{array}{c} x(m,\xi )=\overset{N-1}{\underset{k=0}{\text{∑}}}\left|x_{\text{freq}}(m,k)\right|\cdot f(\xi ,k)\;0\leq m\leq M;\;1\leq \xi \leq 17 \end{array} \end{array}$$

where *f*(*η*,*k*) also represents the weighting factor of the frequency energy at the *k*th point of the *η*th band.

Some undesired impulse noise is resulted from our experiments that the energy *x*(*m*,*η*) obtained in Equation (15). Hence, a three-point median filter is further used to get the smoothed energy, *x̂*(*m*,*ξ*):

(16) $$\hat{x}(m,\xi )=\left[x(m-1,\xi )+x(m,\xi )+x(m+1,\xi )\right]/3.$$

In fact, the noise can focus the same as speech. Based on these finds, we can remove the frequency energy of the beginning interval from the smoothed energy, *x̂*(*m*,*ξ*), to get the pure energy, *X*(*m*,*η*):

(17) $$X(m,\xi )=\hat{x}(m,\xi )-\sum_{j=0}^{4}\hat{x}(j,\xi )/5$$

where $\sum_{j=0}^{4}\hat{x}(j,\xi )/5$ means the frequency energy of the beginning interval estimated by averaging the frequency energy of the first five frames of the recording.

### 3.2. Part-Band Weighting Estimation

We need a parameter will help us know how much the current part-band is corrupted by noise due to the influence of noise upon the detection performance. A posterior part-band SNR, *SNR^pot^*(*m*,*η_p_*) is required in order to determine the part-band utility rate on *η_p_* part for *m*th frame, and it is formulated as:

(18) $${\text{SNR}}^{\text{pot}}(m,\xi_{p})=10\cdot \log_{10}\left[{\text{PBE}}_{N+S}(m,\xi_{p})/{\text{PBE}}_{N}(m,\xi_{p})\right]$$

where *PBE_N_*_+_*_S_*(*m*, *η_p_*) means the part-band energy (PBE) range from on *η_p_* part for *m*th frame for the observed noisy speech signal. *PBE_N_*(*m*, *η_p_*) is the estimated noise part-band energy.

According to Equation (18), we know that the estimated noise part-band energy, *PBE_N_*(*m*, *η_p_*), has to be estimated while determining the value of a posterior SNR, *SNR^pot^*(*m*, *η_p_*). In order to estimate the noise-level quickly and accurately, the method tracking the minimum of the noisy speech power spectrum energy over a fixed search window length was proposed [22]. We use an efficient method [23] to speed up the determination of local minimum of noisy speech spectrum over a search window size, which is not constrained by any window length to update noise spectrum estimate, and it is calculated as below:

(19) $$\begin{array}{l} \text{If}\;{\text{PBE}}_{\min}(m-1,\xi_{p})<{\text{PBE}}_{N+S}(m,\xi_{p}),\\ \text{then}\;{\text{PBE}}_{\min}(m,\xi_{p})=\gamma \cdot {\text{PBE}}_{\min}(m-1,\xi_{p})+\frac{1-\gamma }{1-\beta }\left[{\text{PBE}}_{N+S}(m,\xi_{p})-\beta \cdot {\text{PBE}}_{N+S}(m-1,\xi_{p})\right],\\ \text{else}\;{\text{PBE}}_{\min}(m,\xi_{p})={\text{PBE}}_{N+S}(m,\xi_{p}). \end{array}$$

where *P_min_*(*m*, *η_p_*) denotes the local minimum of power energy of the noisy speech, and it stands for noise part-band energy. *β* and *γ* are experimentally determined constants.

After obtaining the value of a posterior SNR, the part-band weight coefficient, *wef*(*m*, *η_p_*), is calculated by applying a sigmoid function:

(20) $$\text{wef}(m,\xi_{p})=\frac{1}{1+\exp\left[-0.5\cdot \left({\text{SNR}}^{\text{pot}}(m,\xi_{p})-\eta (m,\xi_{p})\right)\right]}$$

where *η* (*m*, *η_p_*) is a center-offset of the sigmoid function, and it is depended on part-band index.

Observing Equation (20), we will use this information to weight each part-band if the *a posteriori* SNR and a center-offset of the sigmoid function are known.

**Figure 7.**
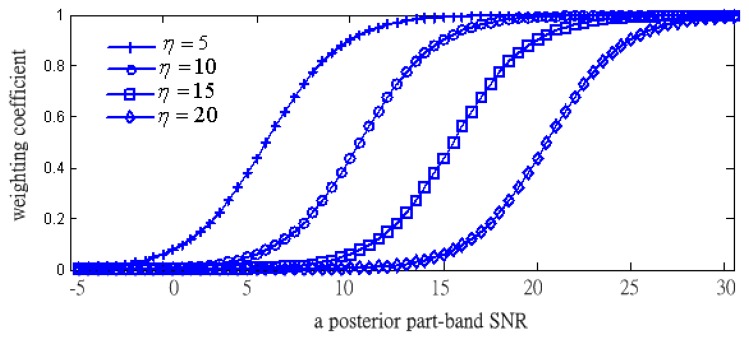
The plots of weight coefficients against a posterior part-band SNR under variable *η*.

Figure 7 shows the plots of the weighting coefficients from Equation (20) depending on *η*. Under the fixed value of a posterior SNR, the weighting coefficients decrease towards zero when *η* is increasing. In addition, the values of all the parameters are determined by experimental tests. According to the fact that the speech components are almost focused in the lower frequency band, we let the sigmoid function with largest *η* (such as *η* = 20) locate to the highest frequency band (such as the HH*th* frequency part). On the contrary, we let the sigmoid function with the smallest *η* (such as *η* = 5) locate to the lowest frequency band (such as LL*th* frequency part).

Consequently, the TD-PBEE parameter can be further weighted and be shown as below:

(21) $$TD-{\text{PBEE}}^{\text{wef}}(m,\xi_{p})=TD-\text{PBEE}(m,\xi_{p})\times \text{wef}(m,\xi_{p})$$

where *TD-PBEE^wef^*(*m*, *η_p_*) denotes the weighted TD-PBEE parameter.

Thus, summing the four TD-PBEEs from each part-band as a combined TD-PBEE, the combined TD-PBEE is expressed as below:

(22) $$TD-{\text{PBEE}}_{\text{comb}}^{\text{wef}}(m)=\sum_{\xi_{p}=LL}^{HH}TD-{\text{PBEE}}^{\text{wef}}(m,\xi_{p})$$

**Figure 8.**
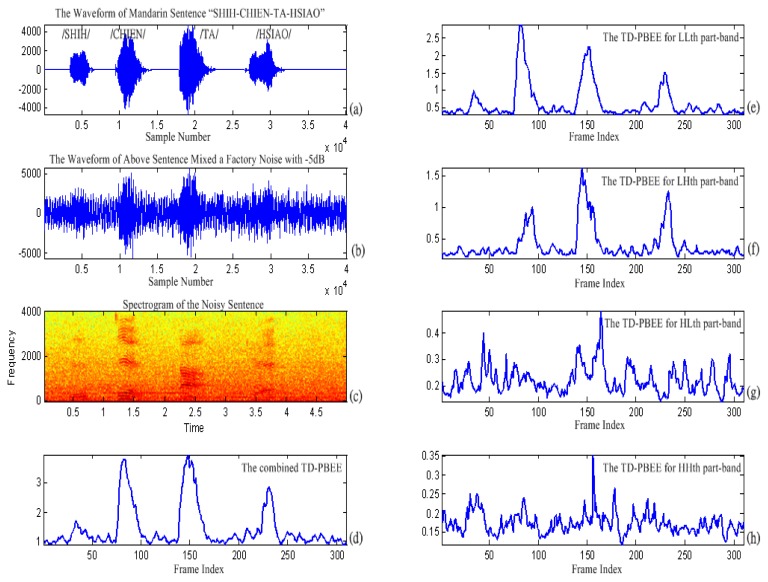
The development of the combined TD-PBEE feature parameter.

Figure 8 shows the results of the combined TD-PBEE compared with TD-PBEE on each part-band. The pronunciation of the Mandarin sentence “SHIH-CHIEN-TA-HSIAO” is diagrammatically shown in Figure 8a. In detail, the waveform of the sentence under factory noise conditions is displayed in Figure 8b. The corresponding spectrogram is also shown in Figure 8c. We find that each TD-PBEE parameter accurately indicates the boundary of voice activity under 5 dB factory noise in Figures 8d–h. We also observe that the combined weighted TD-PBEE summing up the four TD-PBEEs can more accurately extract the voice-activity under 5 dB factory noise conditions than each weighted TD-PBEE.

### 3.3. The VAD Decision

Based on the description of the combined TD-PBEE using short-partition band number *N* and long-term window length *R*, the voice activity is determined by the decision rules as shown below:

(23) $$\begin{array}{l} \text{if}\;(TD-\text{PBEE}(m)>Th_{S})\;\text{VAD}(m)=1;\\ \text{else if}(TD-\text{PBEE}(m)<Th_{N})\;\text{VAD}(m)=0;\\ \text{else}\;\text{VAD}(m)=\text{VAD}(m-1) \end{array}$$

where *Th_s_* and *TH_n_* mean the speech thresholds and noise thresholds, respectively.

The two values can be recursively updated by using the mean and variance of the logarithmic combined TD-PBEE to estimate the time-varying noise characteristics [24]. In fact, we assume that the first four frames only contain noise and then compute the initial noise mean and variance with the first five frames.

The scheme of adaptive threshold for the speech and noise can be computed by the following:

(24) $$Th_{S}=\mu_{N}+\alpha_{S}\cdot \sigma_{N}$$

(25) $$Th_{N}=\mu_{N}+\beta_{N}\cdot \sigma_{N}$$

Similarly, *μ_N_* and *σ_N_* represent the mean and the variance of the logarithmic combined TD-PBEE, respectively. In addition, *α_S_* and *β_N_* are the adjustment constants which are used to determine the threshold.

The mean and variance of the logarithmic combined TD-PBEE are updated while the decision rule shows a noise period:

(26) $$\begin{array}{l} \mu_{N}(m)=\gamma \cdot \mu_{N}(m-1)+(1-\gamma )\cdot TD-\text{PBEE}\\ {\left[TD-{\text{PBEE}}^{2}\right]}_{\text{mean}}(m)=\gamma \cdot {\left[TD-{\text{PBEE}}^{2}\right]}_{\text{mean}}(m-1)+(1-\gamma )\cdot TD-{\text{PBEE}}^{2}\\ \sigma_{N}(m)=\sqrt{{\left[TD-{\text{PBEE}}^{2}\right]}_{\text{mean}}(m)-{\left[\mu_{N}(m)\right]}^{2}} \end{array}$$

where *γ* = 0.5 is chosen by experiment. We then update the threshold using the updated mean and variance of the logarithmic combined TD-PBEE.

## 4. Evaluation and Results

In order to evaluate the proposed TD-PBEE VAD method, the speech database is first described in this section. In addition, the performances of comparison with state-of-the-art VAD algorithms (such as LSFM [13], BSE [19], G.729B [25], AMR2 [26], LTSD [12] and MTED [27]) will be reported as follows.

### 4.1. Database Description

We used a set of 12 sentences (about 107 s) from four different speakers: two males and two females from the TIMIT database to evaluate the advantages of the proposed TD-PBEE feature sets for speech detection. The utterances as speech or non-speech frames are corrupted by four different types of background noise: white noise, factory noise, car noise and babble noise at different average SNR levels ranging between clean and 5 dB (from the NOISEX-92 database). All signals in the database were down-sampled to 8-kHz, mono-channel and 16-bits per sample. In addition, the optimal parameters for the proposed VAD were: *N_LL_* = 8, *N_LH_* = 4, *N_HL_* = 3 and *N_HH_* = 2; *R_LL_* = 5, *R_LH_* = 10, *R_HL_* = 15 and *R_HH_* = 20; *η_HH_* = 5, *η_HL_* = 10, *η_HL_* = 15, and *η_HH_* = 20 while the filter bank decomposed the signal into four part-band from Mel-scaled partition.

### 4.2. The Performance of Comparison with Sate-of-The-Art VAD Algorithms

In order to clearly description the performance of VAD algorithms, the speech/non-speech hit rate (HR1/HR0) as a function of the SNR has been presented in this section. The average speech/non-speech hit rate (HR1/HR0) for each type of noise is employed for comparison between each one and calculated as below:

(27) $$HR0=\frac{\text{number of non}-\text{speech frames correctly classified}}{\text{number of real non}-\text{speech frames}}\times 100\%$$

(28) $$HR1=\frac{\text{number of speech frames correctly classified}}{\text{number of real speech frames}}\times 100\%$$

**Figure 9.**
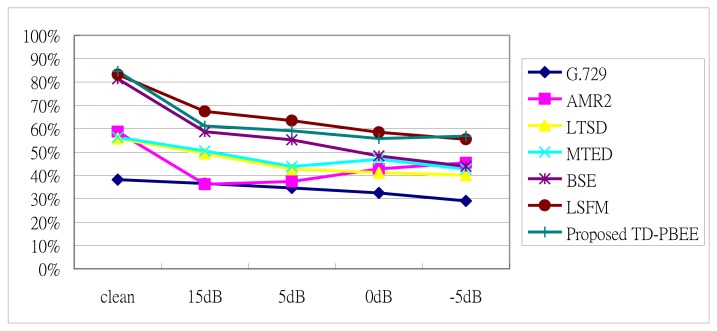
Non-speech hit rate (HR0) from clean to 5 dB under the four types of noise.

**Figure 10.**
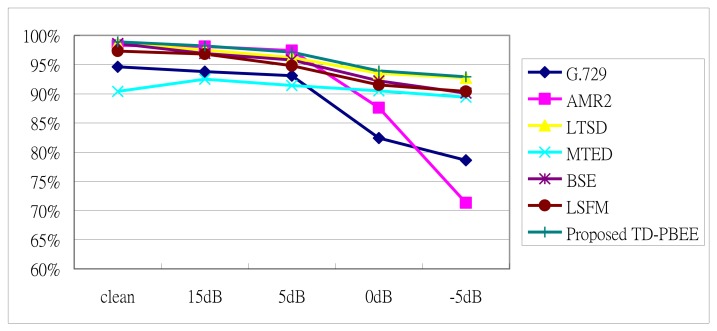
Speech hit rate (HR1) from clean to 5 dB under the four types of noise.

The speech/non-speech hit rate (HR1/HR0) as a function of the SNR for the proposed TD-LTE, G.729, AMR2, LTSD, MTED, BSE and LSFM VAD algorithms are shown in Figure 9 and Figure 10. In these two Figures, we provide the results of non-speech hit rate (HR0) and speech hit rate (HR1), respectively. The results compare the proposed TD-PBEE VAD algorithm to G.729, AMR2, LTSD, MTED, BSE, and LSFM VADs from clean to 5 dB under the four types of noise conditions. We observe that the LSFM VAD is comparable to the proposed TD-PBEE VAD in term of HR0 analysis under lower SNR level. The standard G.729 VAD gives the worst performance among the reference VAD algorithms while performing HR0 analysis. Similarly, we also observe that the LTSD VAD is comparable to the proposed TD-PBEE VAD in terms of HR1 analysis under lower SNR level conditions. In addition, the standard AMR2 VAD has the worst performance among the reference VAD algorithms while performing HR1 analysis at lower SNR level.

In order to further describe the efficiency of VAD for the different types of noises of the NOISEX database, the comparison of performances of VAD algorithms has also been presented in Table 2 and Table 3. We observe that the average accuracy of LSFM VAD is better than the proposed TD-PBEE VAD in Table 2. In detail, the LSFM VAD is superior to the proposed TD-PBEE VAD while testing in factory noise and car noise. However, the LSFM VAD is worse than the proposed TD-PBEE VAD while testing in babble noise. In Table 3, we also observe that the average accuracy of the proposed TD-PBEE VAD is best among all reference VAD algorithms, especially in babble noise. The LTSD is second accuracy of detecting voice. We summarize that the proposed TD-PBEE VAD attains a 63.55% HR0 average value in non-speech detection. Besides, the proposed TD-PBEE VAD also obtains the best behavior in detecting speech with a 96.2% HR1 average value.

Then, the error norm of false alarm rates, *E_norm_*, is used to further quantify the speech/non-speech hit rates, and it is defined as:

(27) $$E_{\text{norm}}=\sqrt{{\left(1-HR1\right)}^{2}+{\left(1-HR0\right)}^{2}}$$

Table 4 shows an average speech/non-speech hit rates (*HR*0 and *HR*1), and overall false error norm (*E_norm_*) for SNR level from clean to 5 dB. We found that the proposed TD-PBEE achieved the minimum false alarm error norm with a 36.65% value and was obviously superior to other VAD algorithms.

## 5. Conclusions

In this paper, we present a novel two-dimensional part-band energy entropy (TD-PBEE) based on short-partition band number *N* and long-term window length *R*. The proposed TD-PBEE-based VAD is composed of four components: Mel-scaled filter bank, TD-PBEE feature extraction, part-band weighting estimation, and the VAD decision. We found that the two-dimensional entropy improves one-dimensional entropy according to the experimental results. We also discussed the estimation of the part-band weighting that can help to understand the useful spectral information of each part-band. We also observed that the optimal parameters: *R* and *N* can increase the accuracy of voice detection. We also performed experiments with the VAD decision, the two thresholds for speech and noise can be updated to detect the speech voice. Future research will apply this to voice-command applications in a realistic environment to increase accuracy.

## Figures and Tables

**Table 1.** The number of part-band partitions related to the VAD accuracy and delay time.

**The Number of Part-Band Partitions**	**VAD Accuracy (%)**	**Delay Time (s)**
2	68.3%	0.64
***4***	***82.3 %***	***0.94***
6	82.5%	1.24
8	83.4%	1.96

**Table 2.** Non-speech hit rate (HR0) from clean to 5 dB under the four types of noise.

**NOISEX Database**	**White**	**Factory**	**Car**	**Babble**	**Average**

**VAD Algorithm**					
**G.729**	71.10%	23.50%	21.30%	20.90%	34.20%
**AMR2**	58.70%	37.40%	37.50%	42.80%	44.10%
**LTSD**	54.30%	43.40%	45.70%	40.20%	45.90%
**MTED**	54.30%	50.40%	43.80%	43.90%	48.10%
**BSE**	81.20%	51.70%	52.20%	44.90%	57.50%
**LSFM**	82.90%	63.20%	61.50%	48.20%	63.95%
***Proposed TD-PBEE***	*83.10%*	*59.70%*	*58.50%*	*52.90%*	*63.55%*

**Table 3.** Speech hit rate (HR1) from clean to 5 dB under the four types of noise.

**NOISEX Database**	**White**	**Factory**	**Car**	**Babble**	**Average**

**NOISEX DatabaseVAD Algorithm**					
**G.729**	89.70%	90.80%	91.10%	82.40%	88.50%
**AMR2**	92.30%	91.70%	92.40%	85.60%	90.50%
**LTSD**	98.60%	95.50%	96.20%	92.50%	95.70%
**MTED**	90.20%	92.50%	91.40%	89.50%	90.90%
**BSE**	97.80%	95.00%	95.10%	90.90%	94.70%
**LSFM**	97.50%	92.30%	93.60%	88.00%	92.85%
***Proposed TD-PBEE***	***98.40%***	***96.10%***	***96.50%***	***93.80%***	***96.20%***

**Table 4.** Average speech/non-speech hit rates and overall false error norm for SNR level from clean to −5 dB.

**VAD**	**G.729**	**AMR2**	**LTSD**	**MTED**	**BSE**	**LSFM**	***Proposed TD-PBEE***

**VADEvaluation**							
**HR1(%)**	88.50%	90.50%	95.70%	90.90%	94.70%	92.85%	*96.20%*
**HR0(%)**	34.20%	44.10%	45.90%	48.10%	57.50%	63.95%	*63.55%*
***Error norm(%)***	66.80%	56.70%	54.27%	52.69%	42.83%	36.75%	***36.65%***
